# Self-perceived burden, fear of progression and psychological flexibility in cervical cancer survivors: A moderated network analysis

**DOI:** 10.1016/j.apjon.2025.100762

**Published:** 2025-07-23

**Authors:** Qihan Zhang, Furong Chen, Zhirui Xiao, Siyu Li, Yiguo Deng, M. Tish Knobf, Jiaying Li, Zengjie Ye

**Affiliations:** a School of Nursing, Guangzhou Medical University, Guangzhou, Guangdong Province, China; b School of Nursing, University of South China, Hengyang, Hunan Province, China; c School of Nursing, Yale University, Orange, CT, United States; d The Nethersole School of Nursing, Faculty of Medicine, The Chinese University of Hong Kong, Hong Kong SAR, China; e School of Nursing, Johns Hopkins University, Baltimore, MD, USA

**Keywords:** Moderated network model, Johnson–Neyman analysis, Psychological flexibility, Self-perceived burden, Fear of progression, Cervical cancer

## Abstract

**Objective:**

Self-perceived burden (SPB) and Fear of progression (FoP) have been reported in people living with cancer and together may increase overall distress. Acceptance and Commitment Theory (ACT) posits that psychological flexibility (PF) may ameliorate the effects of the co-occurrence of these symptoms, yet empirical evidence is scarce. We examined the symptoms associated with SPB and FoP in women with cervical cancer and tested whether PF moderates the association.

**Methods:**

In a cross-sectional study, 307 cervical cancer survivors enrolled in the 2024 “Be Resilient to Cervical Cancer” (BRCC) program completed the Personalized Psychological Flexibility Index (PPFI), the Self-Perceived Burden Scale for Cancer Patients (SPBS-CP), and the Fear of Progression Questionnaire-Short Form (FoP-Q-SF). We constructed symptom-level networks to identify the strongest SPB–FoP connections, and explored the moderating role of PF using Johnson–Neyman analyses.

**Results:**

Among the 307 participants, the mean scores for PPFI were 59.13 (standard deviation [SD] ​= ​10.18), for FoP-Q-SF, 31.12 (SD ​= ​4.98), and for SPBS-CP, 48.18 (SD ​= ​17.12) indicating moderate levels of PF, fear of progression and SPB. Network analysis highlighted the “future worries”- “psychological burden” link as the strongest SPB-FoP link (*β* ​= ​0.34). Johnson–Neyman probing revealed threshold-dependent moderation by PF. PF attenuated one link at high levels (> 84.95) but amplified three links at moderate levels (> 47.51).

**Conclusions:**

PF significantly moderates symptom-level associations between self-perceived burden on fear of progression among cervical cancer survivors. ACT-based interventions may attenuate such association to minimize patients’ psychological distress.

## Introduction

Cervical cancer remains the fourth most common cancer among women worldwide, with over 500,000 new cases each year.^1^ Advances in screening and treatment have lowered mortality, yet survivors continue to face considerable psychological distress.^2^^,^^3^ More than half of them report anxiety and emotional distress following an abnormal screening result and many experience psychological distress during treatment.^4^^,^^5^ Despite receiving standard treatment, over 30% patients with cervical cancer experience disease progression or recurrence.^6^ This inevitably imposes a significant psychological burden on patients across all stages of the disease,^7^ rendering interventions that mitigate the psychological challenges faced by patients indispensable.^8^^,^^9^

Self-perceived burden (SPB) and fear of progression (FoP) are common aspects of psychological distress among cancer survivors.^10^^,^^11^ SPB describes the distress and guilt experienced when patients believe they impose an undue strain on caregivers or family members. SPB is also intensified by worries about harming close relationships and disrupting family roles,^12^ which occurs more frequently among Asian patients with cervical cancer.^13^ Current research suggests that interventions aimed at enhancing psychological flexibility (PF), improving communication, and fostering adaptive coping strategies may help alleviate SPB improving patient outcomes and reducing the psychological burden on patients and their families.^12^ FoP manifests as persistent anxiety about disease recurrence or advancement.^14^^,^^15^

PF is defined as the capacity to maintain present-moment awareness and the ability to engage in valued behaviors despite distressing thoughts or emotions.^16^ Based on the Acceptance and Commitment Theory (ACT), PF represents as a core target in several therapeutic approaches for cancer patients, and has been associated with improved physical and psychological outcomes, informing psychological intervention after diagnosis in cervical cancer treatment.^17^^,^^18^ By improving emotional regulation and promoting values-consistent action, PF enables patients to reframe perceptions of burden and alleviate guilt and inadequacy.^19^

### Theoretical framework

ACT posits that enduring psychological distress arises not from negative emotions per se, but from psychological rigidity described as patterns of avoiding or overidentifying with internal experiences that undermine value-driven action. The central goal of ACT is to promote PF.^20^

Given that SPB and FoP are prevalent among patients with chronic illnesses,^21^^,^^22^ we selected SPB and FoP from negative emotions and employed ACT as the theoretical foundation for understanding the interplay revealing how their interrelationships impact the patient's well-being. FoP manifests as heightened distress from disease progression uncertainty demonstrating high prevalence among advanced-stage cervical cancer patients,^22^^,^^23^ while SPB emerges from cognitive fusion with guilt- inducing narratives. The inherent uncertainty of cancer progression may amplify guilt and anxiety by exacerbating patients' emotional stress in a mutually reinforcing cycle,^24^ suggesting a positive SPB-FoP association. Hence, we propose **Hypothesis 1**: FoP correlates with SPB in cervical cancer patients.

Within the ACT framework, SPB reflects cognitive fusion with guilt-laden self-appraisals, whereas FoP arises from experiential avoidance of uncertainty about disease progression. PF, as the core therapeutic aim of ACT, could intervenes in this cycle reducing patients’ distress through improving emotional regulation and promoting values-based actions.^16^^,^^19^ Therefore, we hypothesize that PF will moderate the strength of the SPB-FoP association, such that greater flexibility weakens their linkage. Then, we propose **Hypothesis 2**: PF plays a significant moderating role in the relationship between FoP and SPB.

However, prior work has not identified interactions among individual symptoms that drive psychological distress in patients with cancer. Symptom-to-symptom (node-level) analysis could address this limitation by revealing which modification of specific SPB and FoP high-centrality nodes are essential to maximally disrupt the SPB-FoP network. Unlike traditional mediation methods,^25^ network analysis is better suited for exploring micro-level or node-specific symptom interactions,^26^ and moderated networks effectively examine moderating variables.^27^ Network analysis can also contribute to detect bridge symptoms that propagate distress between SPB and FoP.^25^^,^^28^ Moderated network models when combined with Johnson–Neyman probing offers two key advantages: they quantify how a continuous moderator (in our study, PF) alters the strength of each symptom–symptom connection across its full range, and they delineate the exact moderator values at which these associations become significant.^27^ This approach thus provides a nuanced, condition-specific map of how PF reshapes the SPB-FoP network.^29^

This study aims to (1) map symptom-level associations between SPB and FoP using moderated network analysis; (2) examine how PF moderates those associations; (3) quantify the regulatory effects of PF on individual SPB-FoP connections via Johnson–Neyman techniques.

## Methods

### Participants

From March to September 2024, we enrolled women with cervical cancer from five tertiary hospitals participating in the “Be Resilient to Cervical Cancer” (BRCC) in Guangdong, Sichuan, and Hunan provinces in China. Eligibility criteria were: female, age ≥ 18 years, ability to understand and communicate in Chinese, and provision of written informed consent. Patients with visual, hearing, cognitive impairments,and mental disorders were excluded. The Ethics Committee of The University of South China approved this study (Approval No. 2023NHHL048).

### Sample size

Network estimation employed a pairwise Markov random field (PMRF) framework, which in our design required 17 node-specific thresholds and 136 pairwise edges (calculated as 17 ​× ​(17–1)/2 ​= ​136),^30^ yielding 153 parameters in total. Following the common guideline of at least one observation per parameter, we determined a base sample requirement of 153 subjects.^31^^,^^32^ To accommodate an anticipated 20% attrition rate, the required sample size was calculated as 192 subjects. Accounting for the Johnson–Neyman analysis requirements, group reapplied Kendall's criterion for sample size determination, yielding a required sample of 255 participants. With a final sample of 307 cervical cancer survivors, our study substantially exceeded this threshold, thereby maximizing the robustness and precision of the network analyses.

### Data collection

#### Demographics and clinical characteristics

Demographic and clinical variables collected included age, nationality, marital status and number of children, household income, educational attainment, employment status, cancer stage and histological type, and current treatment modality.^33^^,^^34^

#### Measurement of FoP

FoP was assessed with 12-item Fear of Progression Questionnaire–Short Form (FoP-Q-SF), recorded on a 1–5 Likert scale (total score range 12–60); a score ≥ 34 indicates clinically significant fear. Cronbach's α in this sample was 0.919. The interpretation of each item was provided in Supplementary Table S1.

#### Measurement of PF

PF was assessed using the 14-item Personalized Psychological Flexibility Index (PPFI), covering avoidance (reverse scored), acceptance, and control.^35^ Items are rated 1 (“strongly disagree”) to 7 (“strongly agree”), with higher totals reflecting greater flexibility. Cronbach's α was 0.84 in this sample.

#### Measurement of SPB

SPB was evaluated by the 21-item Self-Perceived Burden Scale for Cancer Patients (SPBS-CP), comprising caring (items 1–4, dependency in daily activities), family (5–11, treatment-cost guilt and income-loss stress), psychological (12–17, emotional distress from role disruption and lineage anxiety), and treatment (18–21, shame about side effects and caregiving demands) subscales.^36^ Each item uses a 1–5 scale; scores > 30 suggest substantial burden. Cronbach's α was 0.938 in this sample.

### Data analysis

Data analysis covered participants’ demographics, clinical characteristics, network estimation, and Johnson–Neyman probing. All analyses were conducted using SPSS 23.0 and R (v4.4.2).

First, descriptive statistics (means ​± ​standard deviation [SD] for continuous variables; *n* [%] for categorical variables) summarized demographic and clinical data, as well as total and subscale scores of the PPFI, SPBS-CP, and FoP-Q-SF.

Subsequently, moderated network models were constructed using the *modnets* package.^37^ Prior to estimation, nodes were screened by centrality metrics to reduce false positives and improve model interpretability. Node selection was guided by clinical relevance and psychometric validation and all 17 items were retained as nodes. Network structure and moderation effects were simultaneously estimated with the *fitNetwork* function, which employs nodewise (neighbourhood) regression—a method that sequentially fits univariate regression models to infer network connections.^38^^,^^39^

Next, interaction effects and their coefficients were examined using the *plotCoefs* and *intsPlot* functions, enabling precise identification and interpretation of the significant relationships. The network was visualized with *PlotNet* under an ‘AND’ rule, retaining only edges that achieved statistical significance in both nodewise regressions. Analysis employed case-resampling bootstrap methods to evaluate the robustness of the results confirming edge-weight stability.^39^

Finally, significant moderating effects identified in the network were then probed with the Johnson–Neyman technique (*interactions* package),^40^ which delineated the specific range of PF over which each SPB-FoP association remains significant. This combined strategy integrates broad network mapping with granular, threshold-based moderation analysis, offering a detailed view of symptom-level dynamics. JN assumptions were verified: linearity, residual independence, normally distributed residuals, homoscedasticity, and absence of multicollinearity.

## Results

### Participants’ demographics and characteristics

Using convenience sampling, we approached 350 patients; 307 completed the study questionnaires (response rate: 87.7%). Of the 307 participants, the mean age was 42.24 years (SD ​= ​8.62). The majority of women (76.5%) had Stage I or II cancer and the remaining were Stage III or IV. All were receiving treatment Mean scores for the PPFI were 59.1 (SD ​= ​10.2), the FoP-Q-SF, 31.1 (SD ​= ​5.0), and the SPBS-CP, 48.2 (SD ​= ​17.1) indicating moderate levels of PF, fear of progression and SPB. Further demographic and clinical details are presented in Table 1.

**Table 1:** Participants’ demographic and clinical characteristics (*N* ​= ​307).

Variables	Data
**Age (years, mean** ​**± ​standard deviation)**	42.24 ​± ​8.62
**Marital status**	
Unmarried	33 (10.7%)
Married	216 (70.4%)
Other (e.g., divorced, widowed)	58 (18.9%)
**Number of children**	
None	42 (13.7%)
One	158 (51.5%)
Two	85 (27.7%)
More than two	22 (7.2%)
**Place of residence**	
Urban	146 (47.6%)
Rural	161 (52.4%)
**Medical insurance type**	
Social insurance	241 (78.5%)
Business insurance	45 (14.7%)
Uninsured	21 (6.8%)
**Household income** (CNY per person per month)	
< ​3000	24 (7.8%)
3000-4999	163 (53.1%)
> ​5000	117 (38.1%)
**Educational attainment**	
Primary school and below	53 (17.3%)
Junior high school	84 (27.4%)
Senior high school	78 (25.4%)
Undergraduate	61 (19.9%)
Postgraduate and above	31 (10.1%)
**Employment status**	
Unemployed or retired	57 (18.6%)
Employed	250 (81.4%)
**Cancer stage**	
I	56 (18.2%)
II	179 (58.3%)
III	51 (16.6%)
IV	21 (6.8%)
**Histological type**	
Squamous carcinoma	236 (76.8%)
Adenocarcinoma	71 (23.1%)
**Current treatment modality**	
Chemotherapy only	43 (14.0%)
Radiotherapy only	23 (7.5%)
Combined chemotherapy and radiotherapy	67 (21.8%)
Surgery only	75 (24.4%)
Surgery plus chemotherapy	73 (23.8%)
Surgery plus radiotherapy	26 (8.5%)

### Moderated network model

**Fig. 1:**
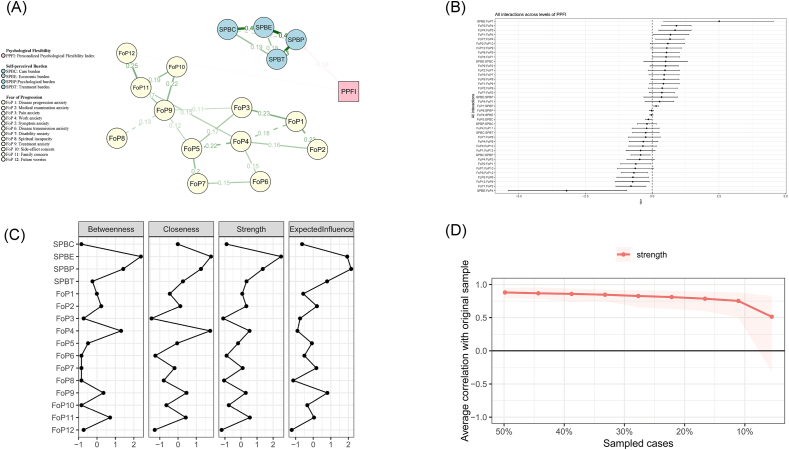
Moderated network analysis visualization. (A): Moderated network analysis visualization. (Moderated connections: “spiritual incapacity” and “treatment anxiety”; “work anxiety” and “disease progression anxiety”; “work anxiety and symptom anxiety”; “disease progression anxiety” and “medical examination anxiety”; “care burden” and “psychological burden”). (B): The 95% confidence intervals of all interactions. (C): Centrality Plot. (D): Average correlation with original sample.

Variable selection and nodewise adjacency matrices are provided in Supplementary Table S2 and S3. Supplementary Fig. S1 and Fig. 1B display regression coefficients with 95% confidence intervals (CIs) for all predictor variables. Specifically, Supplementary Fig. S1 systematically displays the standardized regression coefficients across 16 distinct subFig.s, with each subFig. representing an individual predictor variable. Fig. 1B illustrates the interaction effect sizes and 95% CIs across varying levels of PF. In the estimated moderated network model, the most central symptoms (highest expected influence, EI) were “psychological burden” (EI ​= ​2.16), “economic burden” (EI ​= ​1.93), and “treatment anxiety” (EI ​= ​0.80). Definitions for all symptoms are provided in Supplementary Table S1.

PF significantly moderated multiple symptom-to-symptom connections (Fig. 1A), including those between “spiritual incapacity” and “treatment anxiety”; “work anxiety” and “disease progression anxiety”; “work anxiety and symptom anxiety”; “disease progression anxiety” and “medical examination anxiety”; and “care burden” and “psychological burden”. Among SPB-FoP connections, the strongest associations were between “future worries” and “psychological burden” (*β* ​= ​0.34), “treatment burden” and “psychological burden” (*β* ​= ​0.33), “economic burden” and “psychological burden” (*β* ​= ​0.32), and “family concern” and “psychological burden” (*β* ​= ​0.29). Edge-weight stability was confirmed via case-dropping bootstrap (Fig. 1C and D).

### Johnson–Neyman moderation analysis

**Fig. 2:**
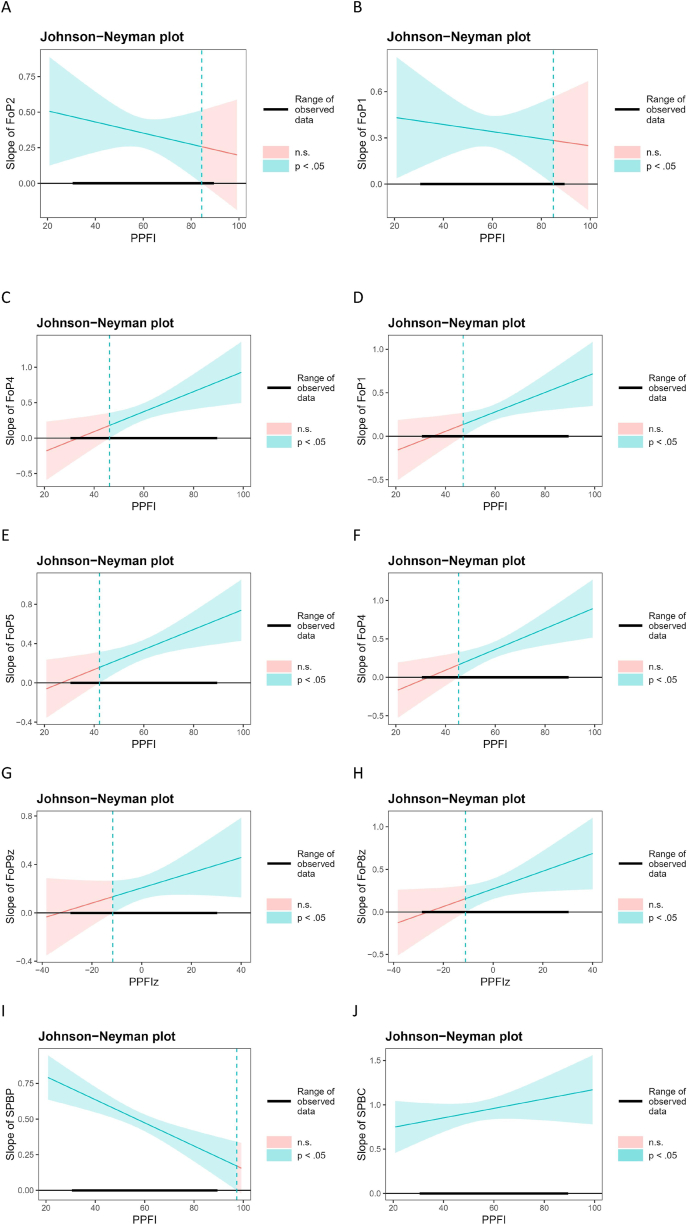
Johnson–Neyman plots. A: Estimated effect of “Disease progression anxiety” (FoP1) ​× ​PPFI on “Medical examination anxiety” (FoP2). B: Estimated effect of “Medical examination anxiety” (FoP2) ​× ​PPFI on “Disease progression anxiety” (FoP1). C: Estimated effect of “Disease progression anxiety” (FoP1) ​× ​PPFI on “Work anxiety” (FoP4). D: Estimated effect of “Work anxiety” (FoP4) ​× ​PPFI on “Disease progression anxiety” (FoP1). E: Estimated effect of “Work anxiety” (FoP4) ​× ​PPFI on “Symptom anxiety” (FoP5). F: Estimated effect of “Symptom anxiety” (FoP5) ​× ​PPFI “Work anxiety” (FoP4). G: Estimated effect of “Spiritual incapacity” (FoP8) ​× ​PPFI on “Treatment anxiety” (FoP9). H: Estimated effect of “Treatment anxiety” (FoP9) ​× ​PPFI on “Spiritual incapacity” (FoP8). I: Estimated effect of “Care burden” (SPBC) ​× ​PPFI on “Psychological burden” (SPBP). J: Estimated effect of “Psychological burden” (SPBP) ​× ​PPFI on “Care burden” (SPBC). FoP, Fear of progression; PPFI, Personalized Psychological Flexibility Index; SPB, Self-perceived burden.

Johnson–Neyman plots (Fig. 2) clarified the effects of PF on key associations, between “disease progression anxiety” and “medical examination anxiety”, “disease progression anxiety” and “work anxiety”, “work anxiety” and “symptom anxiety”, “spiritual incapacity” and “treatment anxiety”, as well as “psychological burden” and “care burden” (Fig. 2).

Notably, high PF (> 84.95) weakens the association between “disease progression anxiety” and “medical examination anxiety” (Fig. 2A and B) while high PF exacerbates the association between “disease progression anxiety” and “work anxiety” (> 46.20, Fig. 2C and D), “work anxiety” and “symptom anxiety” (> 42.16, Fig. 2E and F), “treatment anxiety” and “spiritual incapacity” (> 47.51, Fig. 2G and H). As for the association between “psychological burden” and “care burden”, no cut-offs of PF are recognized (Fig. 2I and J).

## Discussion

This study employed a moderated network approach to elucidate how PF shapes the interplay between symptoms of FoP and SPB in cervical cancer survivors Our findings confirm that PF significantly moderates key symptom-to-symptom links within the SPB-FoP network, offering novel, mechanistic insights that extend. Although our findings demonstrated that PF moderated inter-symptom relationships, this did not directly imply that enhancing PF would reduce symptom severity. The analysis revealed that PF influenced the strength of symptom–symptom associations, not the absolute severity levels. Therefore, the more accurate and cautious interpretation is: Interventions tailored to individual PF levels might optimize treatment efficacy, rather than assuming PF enhancement alone directly reduces symptoms.

We identified four SPB-FoP connections of greatest clinical relevance: future worries - psychological burden, treatment burden - psychological burden, economic burden - psychological burden, and family concern - psychological burden. These results support Hypothesis 1 and the association of 10.13039/100004931SPB and FoP, demonstrating that persistent worry about the future and tangible stressors, particularly financial strain, intensify patients’ psychological burden.^15^^,^^41^

This aligns with established evidence that economic hardship amplifies cancer-related anxiety and corrodes quality of life independently of psychological factors.^42^, ^43^, ^44^ Our results therefore underscore a dual imperative for biopsychosocial care: First, oncology clinics must implement integrated screening protocols that concurrently assess financial distress and psychosocial vulnerability during routine visits. Second, for modifiable pathways like the moderated treatment burden-psychological burden link, embedding brief, values-based interventions can disrupt distress cycles. Critically, the unmoderated economic burden pathway demands policy-level action: Expanding insurance coverage for mental health services, subsidizing out-of-pocket treatment costs, and mandating patient navigation programs are essential to mitigate burdens that lie beyond individual psychological coping. Only through such tiered approaches—addressing both moderated symptom networks through psychological support and unmoderated socioeconomic drivers through structural interventions—can holistic patient care be achieved.^45^

Second, our findings show that PF dynamically moderates the bidirectional association between SPB and FoP, confirming Hypothesis 2. By targeting psychological rigidity, interventions aimed at enhancing PF may disrupt the pathological feedback loop between SPB and FoP. These results underscore PF as a key moderator in this relationship, highlighting its role as a complex, non-linear mechanism for managing psychological distress in cancer care. The moderated links often represent theorized vicious cycles that are key drivers of distress within disorders, even if they are not the strongest between-community connections. PF may not always weaken the strongest symptom associations but could empower individuals to engage in values-based action despite these associations, thereby reducing functional impairment and potentially mitigating the overall impact of the network. We link this interpretation to the established efficacy of PF-targeted therapies like ACT, which reliably reduce global symptom severity and improve functioning. PF's core action may lie in reducing the distress and functional impact associated with symptoms and their co-occurrence (e.g., via defusion, acceptance), rather than necessarily altering the direct symptom–symptom associations themselves. At low levels of PF, we observed a buffering effect whereby increases in PF attenuated the impact of disease progression anxiety on medical examination anxiety, effectively weakening their linkage. This supports prior evidence suggesting that PF modulates anxiety responses in clinical settings.^46^ Additionally, under conditions of limited PF, a negative association between care burden and psychological burden emerged, suggesting that improving care quality may reduce psychological distress even when patients' PF is compromised.^47^, ^48^, ^49^

Conversely, at higher levels of PF, we observed an unexpected amplification effect: the positive associations between disease-progression anxiety and work anxiety, and between treatment anxiety and spiritual incapacity, became stronger as PF increased. We interpret this counterintuitive finding as a context-dependent phenomenon requiring cautious speculation. This may suggests that, although PF is generally adaptive, it may under certain conditions heighten the salience of specific stressors.^50^ Elevated PF appears to raise individuals’ threshold for conscious stress responses, thereby amplifying awareness of symptom-anxiety linkages. Such a pattern tentatively aligns with the vigilance-avoidance model of anxiety regulation, which posits that adaptive coping strategies can initially increase threat detection before facilitating avoidance.^51^^,^^52^

### Implications for nursing practice and research

In clinical settings, the interplay among FoP, SPB, and PF underscores the potential of incorporating PF into comprehensive cervical cancer care. It may suggest that clinical practitioners could clinicians could establish scheduled on-site ACT workshops to systematically enhance PF. These workshops could include role-playing to address SPB and deliver psychoeducation on PF.^53^ Clinically, our results support stratified interventions based on patients' current PF levels. For patients with reduced PF, interventions such as ACT that aim to enhance PF to optimal levels may simultaneously alleviate FoP and SPB, supporting a more integrated approach to psychological adaptation and improved treatment outcomes. Consider disrupting strong SPB-FOP symptom connections through exposure therapy and cognitive defusion techniques which inherently improve PF. In contrast, for patients exhibiting elevated PF, clinicians should consider prioritizing health education and cognitive reframing strategies to address maladaptive appraisals. This approach can help patients gain a more accurate understanding of their condition and reduce the risk of excessive psychological burden. Future research is warranted to identify dynamic thresholds of PF that can inform personalized psychological interventions. Integrating PF into cancer care could facilitate more holistic support, enhancing both quality of life and treatment efficacy.^54^^,^^55^

### Limitations

Limitations of this study should be acknowledged. First, the cross-sectional design would need longitudinal studies to explore the temporal dynamics among PF, FoP, and SPB. Also, PF is a relatively stable trait-like construct while FoP represents a fluctuating state variable, our findings might be influenced by the timing of data collection. Second, the sample consisted exclusively of Chinese patients diagnosed with cervical cancer, which may limit the generalizability of findings to other populations and cancer types. Third, this study relies entirely on self-report measures, raising concerns about common method bias. Future studies should consider incorporating clinician-rated or behavioral measures. Finally, this study did not consider potential confounding factors such as cancer stage and treatment modality, which may also affect the accuracy of report.

## Conclusions

This study elucidates the dual regulatory role of PF in moderating FoP and SPB among cervical cancer patients. The findings suggest a need for targeted intervention strategies based on PF levels. Individuals with lower PF may benefit from interventions to build adaptive capacity, while those with heightened PF may require support to prevent hypervigilance and maladaptive cognitive sensitivity.

## CRediT authorship contribution statement

**Qihan Zhang:** Data curation, Software and writing original draft. **Furong Chen:** Data curation, Methodology, Writing – review & editing and validation. **Zhirui Xiao**: Investigation, Software. **Siyu Li and Yiguo Deng**: Investigation, Writing – review & editing. **M. Tish Knobf, Jiaying Li and Zengjie Ye:** Supervision, Writing – review & editing. All authors read and approved the final manuscript.

## Ethical statement

This study was performed in line with the principles of the Declaration of Helsinki. Approval was granted by the Ethics Committee of The University of South China (Approval No. 2023NHHL048). All participants provided written informed consent.

## Data availability statement

Data supporting the findings of this study are available from the corresponding author, ZY, upon request.

## Declaration of generative AI and AI-assisted technologies in the writing process

No AI tools/services were used during the preparation of this work.

## Funding

This research was funded by grants from the National Natural Science Foundation of China (Grant Nos. 72274043, 71904033), the Young Elite Scientists Sponsorship Program by CACM (Grant No. 2021-QNRC2-B08), Guangdong Philosophy and Social Science Foundation (Grant No. GD25YSH17), and Sanming Project of Medicine in Shenzhen (Grant No. SZZYSM202206014). The funders had no role in considering the study design or in the collection, analysis, interpretation of data, writing of the report, or decision to submit the article for publication.

## Declaration of competing interest

The authors declare no conflict of interest. Professor Zengjie Ye, the corresponding author, serves on the editorial board of the *Asia–Pacific Journal of Oncology Nursing*. The article underwent standard review procedures of the journal, with peer review conducted independently of Professor Ye and their research groups.
