# Supplementary material for: Self-perceived burden, fear of progression and psychological flexibility in cervical cancer survivors: A moderated network analysis
Source: Asia Pac J Oncol Nurs. 2025 Jul 23;12:100762. doi: 10.1016/j.apjon.2025.100762 (PMC12355130; doi:10.1016/j.apjon.2025.100762)

Table S1. Node abbreviation index

| **Node** | **Description** | **ltem** | **Scale** |
| --- | --- | --- | --- |
| **FoP 1** | **Disease progression anxiety** | **Being afraid of disease progression** | **FoP-Q-SF** |
| **FoP 2** | **Medical examination anxiety** | **Being nervous prior to doctors’ appointments or periodic examinations** |
| **FoP 3** | **Pain anxiety** | **Being afraid of pain** |
| **FoP 4** | **Work anxiety** | **Being afraid of becoming less productive at work** |
| **FoP 5** | **Symptom anxiety** | **Having physical symptoms, e.g. rapid heartbeat, stomach ache, nervousness** |
| **FoP 6** | **Disease transmission anxiety** | **Being afraid by the possibility that the children could contract cancer** |
| **FoP 7** | **Disability anxiety** | **Being afraid of relying on strangers for activities of daily living** |
| **FoP 8** | **Spiritual incapacity** | **Being afraid of no longer be able to pursue hobbies** |
| **FoP 9** | **Treatment anxiety** | **Being afraid of severe medical treatments in course of the illness** |
| **FoP 10** | **Side-effect concern** | **Worrying that medications could damage the body** |
| **FoP 11** | **Family concern** | **Worrying about what will become of the family if something should happen to the patient** |
| **FoP 12** | **Future worries** | **Being afraid of not being able to work anymore** |
| **SPBC** | **Care burden** | **The burden of care experienced by family caregivers of patients with chronic illnesses** | **SPBS-CP** |
| **SPBE** | **Economic burden** | **The economic burden associated with chronic disease management and healthcare expenditures** |
| **SPBP** | **Psychological burden** | **The psychological burden associated with chronic illness management and its impact on quality of life** |
| **SPBT** | **Treatment burden** | **The socioeconomic and psychological treatment burden imposed by long-term therapeutic interventions** |
| **PPFI** | **Personalized Psychological Flexibility Index** | **The multidimensional index quantifying personalized capacity for cognitive-behavioral adaptation in varying circumstances** | **PPFI** |

Table S2. Variable selection results through varSelect function

|  | SPBC | SPBE | SPBP | SPBT | FoP1 | FoP2 | FoP3 | FoP4 | FoP5 | FoP6 | FoP7 | FoP8 | FoP9 | FoP10 | FoP11 | FoP12 |
| --- | --- | --- | --- | --- | --- | --- | --- | --- | --- | --- | --- | --- | --- | --- | --- | --- |
| mods | SPBE | SPBC | SPBC | SPBC | SPBC | SPBC | FoP1 | SPBC | SPBC | SPBC | SPBT | SPBP | FoP2 | SPBE | SPBP | SPBE |
|  | SPBP | SPBP | SPBE | SPBE | SPBP | SPBE | FoP2 | SPBE | FoP2 | SPBP | FoP1 | FoP2 | FoP3 | SPBP | SPBT | SPBP |
|  | SPBT | SPBT | SPBT | SPBP | SPBT | FoP1 | FoP5 | FoP1 | FoP3 | SPBT | FoP2 | FoP3 | FoP5 | FoP1 | FoP4 | SPBT |
|  | PPFI | FoP2 | FoP8 | FoP9 | FoP2 | FoP3 | FoP7 | FoP2 | FoP4 | FoP4 | FoP3 | FoP6 | FoP6 | FoP6 | FoP9 | FoP5 |
|  |  | FOP4 | FoP11 | FoP12 | FoP3 | FoP4 | FoP9 | FoP3 | FoP6 | FoP5 | FoP4 | FoP7 | FoP8 | FoP7 | FoP10 | FoP6 |
|  |  | FoP7 | PPFI |  | FoP4 | FoP5 |  | FoP5 | FoP7 | FoP7 | FoP5 | FoP9 | FoP10 | FoP8 | FoP12 | FoP7 |
|  |  | FOP12 |  |  | FoP6 | FoP7 |  | FoP6 | FoP9 | FoP8 | FoP6 | FoP10 | FoP11 | FoP9 |  | FoP8 |
|  |  | PPFI |  |  | FoP7 | FoP8 |  | FoP7 | FoP11 | FoP9 | FoP8 | FoP12 | PPFI | FoP11 |  | FoP9 |
|  |  |  |  |  | FoP8 | FoP9 |  | FoP9 | FoP12 | FoP10 | FoP10 | PPFI |  |  |  | FoP11 |
|  |  |  |  |  | FoP9 | FoP10 |  | FoP10 | PPFI | FoP12 | FoP11 |  |  |  |  | PPFI |
|  |  |  |  |  | FoP10 | FoP11 |  | FoP11 |  |  | FoP12 |  |  |  |  |  |
|  |  |  |  |  | FoP11 | PPFI |  | PPFI |  |  | PPFI |  |  |  |  |  |
|  |  |  |  |  | FoP12 |  |  |  |  |  |  |  |  |  |  |  |
|  |  |  |  |  | PPFI |  |  |  |  |  |  |  |  |  |  |  |
| ints | SPBP:PPFI | SPBC:PPFI | SPBC:PPFI |  | SPBP:PPFI | FoP1:PPFI |  | SPBE:PPFI | SPBC:PPFI |  | FoP2:PPFI | SPBP:PPFI | FoP2:PPFI |  |  | FoP8:PPFI |
|  | SPBT:PPFI | SPBP:PPFI |  |  | FOP2:PPFI | FoP7:PPFI |  | FoP1:PPFI | FoP4:PPFI |  | FoP4:PPFI | FoP9:PPFI | FoP8:PPFI |  |  | FoP9:PPFI |
|  |  | FoP4:PPFI |  |  | FOP4:PPFI | FoP9:PPFI |  | FoP3:PPFI | FoP6:PPFI |  |  | FoP12:PPFI |  |  |  |  |
|  |  | FoP7:PPFI |  |  | FOP8:PPFI | FoP10:PPFI |  | FoP5:PPFI | FoP7:PPFI |  |  |  |  |  |  |  |
|  |  |  |  |  | FOP9:PPFI |  |  | FoP7:PPFI |  |  |  |  |  |  |  |  |
|  |  |  |  |  | FOP10:PPFI |  |  | FoP9:PPFI |  |  |  |  |  |  |  |  |
|  |  |  |  |  | FOP12:PPFI |  |  | FoP10:PPFI |  |  |  |  |  |  |  |  |
|  |  |  |  |  |  |  |  | FoP11:PPFI |  |  |  |  |  |  |  |  |

Table S3. The adjacency matrix of moderated network

|  | V1 | V2 | V3 | V4 | V5 | V6 | V7 | V8 | V9 | V10 | V11 | V12 | V13 | V14 | V15 | V16 | V17 |
| --- | --- | --- | --- | --- | --- | --- | --- | --- | --- | --- | --- | --- | --- | --- | --- | --- | --- |
| 1 | 0 | 0 | 0 | 0 | 0 | 0 | 0 | 0 | 0 | 0 | 0 | 0 | 0 | 0 | 0 | 0 | 0 |
| 2 | 0 | 0 | 0.305 | 0.117 | 0.136 | 0 | 0 | 0 | 0 | 0 | 0 | 0 | 0 | 0 | 0 | 0 | 0 |
| 3 | 0 | 0.305 | 0 | 0.413 | 0.159 | 0 | 0 | 0 | 0 | 0 | 0 | 0 | 0 | 0 | 0 | 0 | 0 |
| 4 | 0 | 0.117 | 0.413 | 0 | 0.392 | 0 | 0 | 0 | 0 | 0 | 0 | 0 | 0 | 0 | 0 | 0 | 0 |
| 5 | 0 | 0.136 | 0.159 | 0.392 | 0 | 0 | 0 | 0 | 0 | 0 | 0 | 0 | 0 | 0 | 0 | 0 | 0 |
| 6 | 0 | 0 | 0 | 0 | 0 | 0 | 0.138 | 0.140 | 0.109 | 0 | 0 | 0 | 0 | 0 | 0 | 0 | 0 |
| 7 | 0 | 0 | 0 | 0 | 0 | 0.138 | 0 | 0 | 0.053 | 0 | 0 | 0 | 0 | 0 | 0 | 0 | 0 |
| 8 | 0 | 0 | 0 | 0 | 0 | 0.140 | 0 | 0 | 0 | 0.081 | 0 | 0 | 0 | 0 | 0 | 0 | 0 |
| 9 | 0 | 0 | 0 | 0 | 0 | 0.109 | 0.053 | 0 | 0 | 0.139 | 0 | 0 | 0 | 0 | 0 | 0.073 | 0 |
| 10 | 0 | 0 | 0 | 0 | 0 | 0 | 0 | 0.081 | 0.139 | 0 | 0 | 0.121 | 0 | 0 | 0 | 0 | 0 |
| 11 | 0 | 0 | 0 | 0 | 0 | 0 | 0 | 0 | 0 | 0 | 0 | 0 | 0 | 0 | 0 | 0 | 0 |
| 12 | 0 | 0 | 0 | 0 | 0 | 0 | 0 | 0 | 0 | 0.121 | 0 | 0 | 0 | 0 | 0 | 0 | 0 |
| 13 | 0 | 0 | 0 | 0 | 0 | 0 | 0 | 0 | 0 | 0 | 0 | 0 | 0 | 0 | 0 | 0 | 0 |
| 14 | 0 | 0 | 0 | 0 | 0 | 0 | 0 | 0 | 0 | 0 | 0 | 0 | 0 | 0 | 0.130 | 0.085 | 0 |
| 15 | 0 | 0 | 0 | 0 | 0 | 0 | 0 | 0 | 0 | 0 | 0 | 0 | 0 | 0.130 | 0 | 0.129 | 0 |
| 16 | 0 | 0 | 0 | 0 | 0 | 0 | 0 | 0 | 0.074 | 0 | 0 | 0 | 0 | 0.085 | 0.129 | 0 | 0.192 |
| 17 | 0 | 0 | 0 | 0 | 0 | 0 | 0 | 0 | 0 | 0 | 0 | 0 | 0 | 0 | 0 | 0.192 | 0 |

Figure S1. Plot displaying estimated model coefficients and confidence intervals.


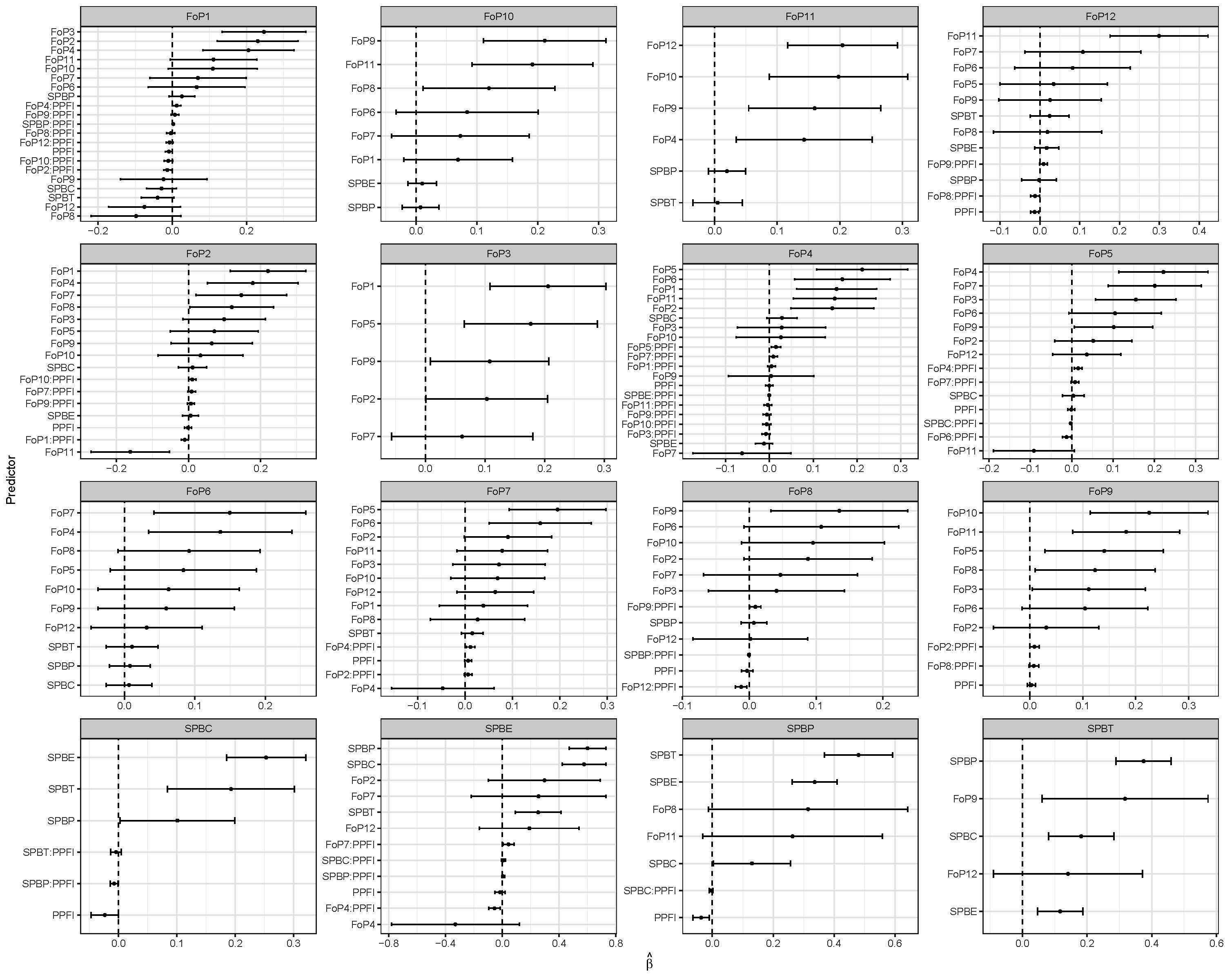

Supplement: Multimedia component 1 [file mmc1.doc]
